# Endoscopic Management of Laryngopyoceles: A Report of Two Cases

**DOI:** 10.7759/cureus.116375

**Published:** 2026-09-16

**Authors:** Leon Yeung, Luke C Wang, Dan Gordon

**Affiliations:** 1 Department of Otolaryngology, Monash Health, Melbourne, AUS

**Keywords:** acute airway obstruction, endoscopic surgery, laryngocele, laryngopyocele, supraglottic mass

## Abstract

Laryngoceles are rare pathological air-filled dilatations of the laryngeal saccule. Rarely, laryngoceles can become infected and pus-filled, forming laryngopyoceles. If untreated, they can lead to life-threatening complications including airway obstruction and deep neck space sepsis. The generally accepted management of laryngopyoceles is surgical, but there are no consensus guidelines available.

We present two cases of elderly patients (89- and 90-year-old) with laryngopyoceles who were successfully managed with endoscopic laryngeal surgery. Both presented with worsening airway symptoms on the background of progressive dysphonia. Fibreoptic nasal laryngoscopy showed large supraglottic masses/swellings, and they underwent emergent microlaryngoscopy and deroofing of an infected laryngocele. Airway management was shared between the ENT and anaesthetics teams. One patient had awake fibreoptic intubation, while the other was trialled with apnoeic/Optiflow and insertion of a microlaryngeal tube via an operative laryngoscope, but required tracheostomy due to limited access. They recovered well in the hospital with intravenous dexamethasone and antibiotics. Both patients were asymptomatic at the one-month follow-up, with no laryngocele recurrence on repeat flexible nasendoscopy. Histopathology did not show any malignancy.

The rarity of laryngopyoceles highlights the importance of maintaining a high index of suspicion in patients presenting with signs of airway compromise or potential deep neck space infection. Suspected cases should usually undergo prompt CT and flexible nasal laryngoscopy evaluation, with early ENT referral. Consideration should also be given to the most suitable approach to managing the airway and the surgical approach to drainage and excision. Early recognition and definitive management of laryngopyoceles are important to prevent potentially life-threatening complications.

## Introduction

Laryngoceles are uncommon pathological air-filled dilatations or herniations of the laryngeal saccule (the appendix of the laryngeal ventricle of Morgagni), which is usually situated between the ventricular folds, the base of the epiglottis, and the inner surface of the thyroid cartilage. They can be congenital or acquired and can be classified as internal, external, or mixed based on whether they are confined to the larynx or whether they herniate through the thyrohyoid membrane. A rare complication is when they become infected and pus-filled, forming laryngopyoceles. Approximately 8% of laryngoceles become infected, and there have been only 64 reported cases in the literature of laryngopyoceles from 1925 to 2019 [1-5]. If untreated, they can lead to progressive inflammation, oedema, or spontaneous rupture, causing purulent discharge into the airways or parapharyngeal spaces. This may result in life-threatening complications including airway obstruction, aspiration, jugular venous thrombosis or mediastinal abscess. While the generally accepted management of laryngopyoceles is surgical, such as an endoscopic approach to decompression via microlaryngoscopy, there are no consensus guidelines available. Therefore, we report on two patients with laryngopyoceles who were successfully managed with endoscopic laryngeal surgery and aim to add to and summarise the current sparse literature for the workup and management of laryngopyoceles.

This article was previously presented as a meeting abstract at the 2026 Royal Australasian College of Surgeons’ Annual Scientific Congress on May 1, 2026.

## Case presentation

This study is reported according to the AME Case Series reporting checklist (adapted from CARE and PROCESS checklists and guidelines). The patients provided informed consent for their de-identified medical information to be used. Herein, we report two cases of laryngopyoceles which were successfully managed with micro-laryngeal surgery.

Case 1

A 90-year-old female patient presented to the emergency department at our tertiary centre with four days of significant dysphonia, odynophagia, stridor at rest, and right-sided otalgia. She was unable to lie flat and was waking up from sleep gasping and choking. This was on the background of six months of progressive dysphonia, as well as increasing dysphagia and odynophagia in the last month. An external MRI done in the community with an otolaryngologist had shown a right supraglottic mucocele. Her pertinent past medical history included gastro-oesophageal reflux disease, hypertension, and hypercholesterolaemia. She was otherwise functionally independent from home alone.

On examination in the emergency department, she required two litres of supplemental oxygen via low-flow nasal prongs, with SpO2 of 93% and respiratory rate of 23 breaths per minute. She was otherwise afebrile and haemodynamically stable with a blood pressure of 143/90mmHg and heart rate of 82 beats per minute. She had some quiet stridor at rest, which improved with intravenous dexamethasone. Flexible nasendoscopy (FNE) demonstrated a large smooth-walled swelling in the right supraglottis obstructing 75% of the view of the glottis. 

Laboratory results were unremarkable, with normal inflammatory markers. She was commenced on intravenous ceftriaxone and metronidazole. CT neck demonstrated a 3.6cm right-sided, fluid-filled internal laryngocele (Figures 1, 2). There was no extension through the thyrohyoid membrane, and it caused approximately 75% narrowing of the airway. There was also no lymphadenopathy. 

**Figure 1:**
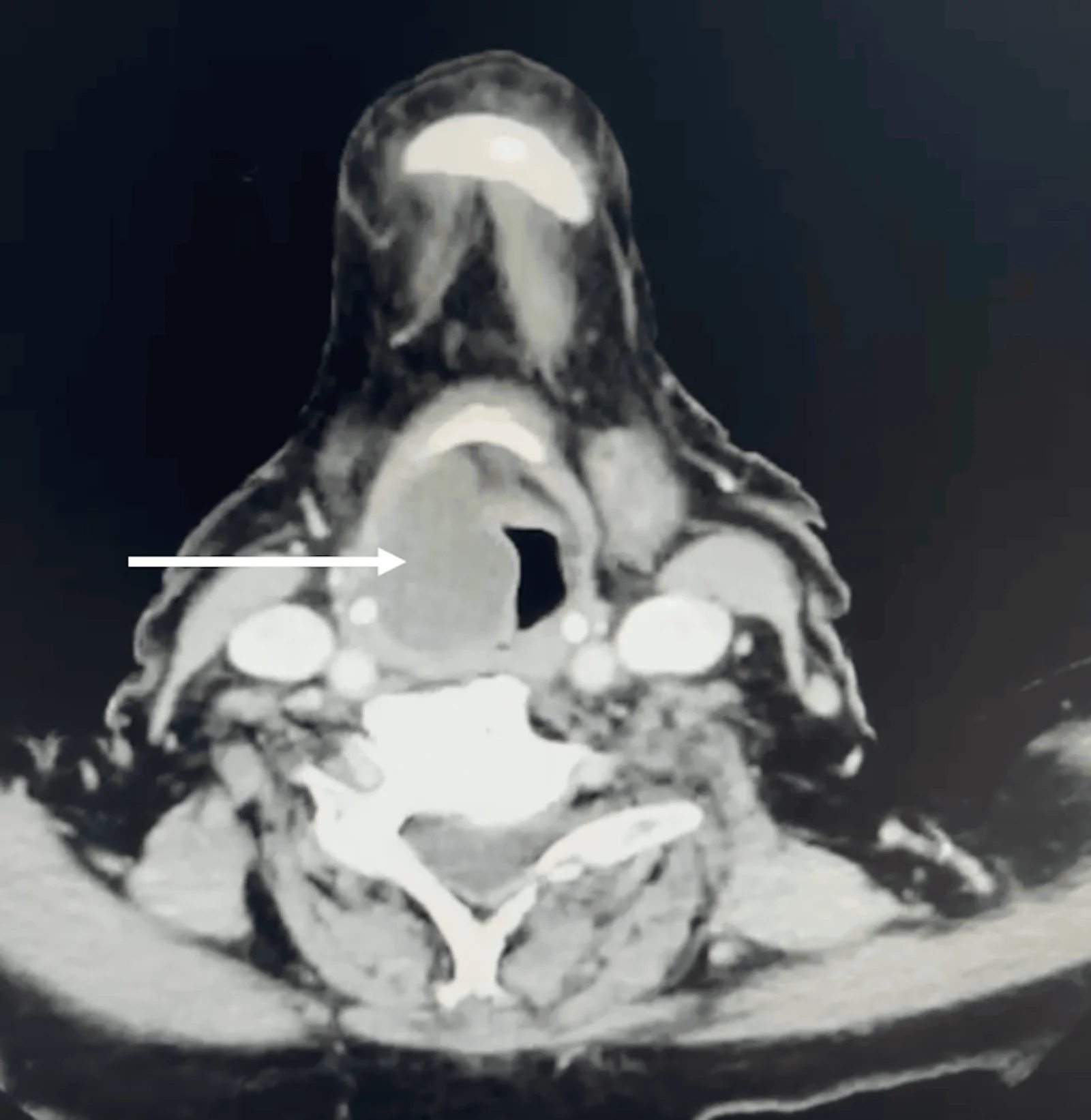
Axial CT of the neck demonstrating a right-sided, fluid-filled internal laryngocele

**Figure 2:**
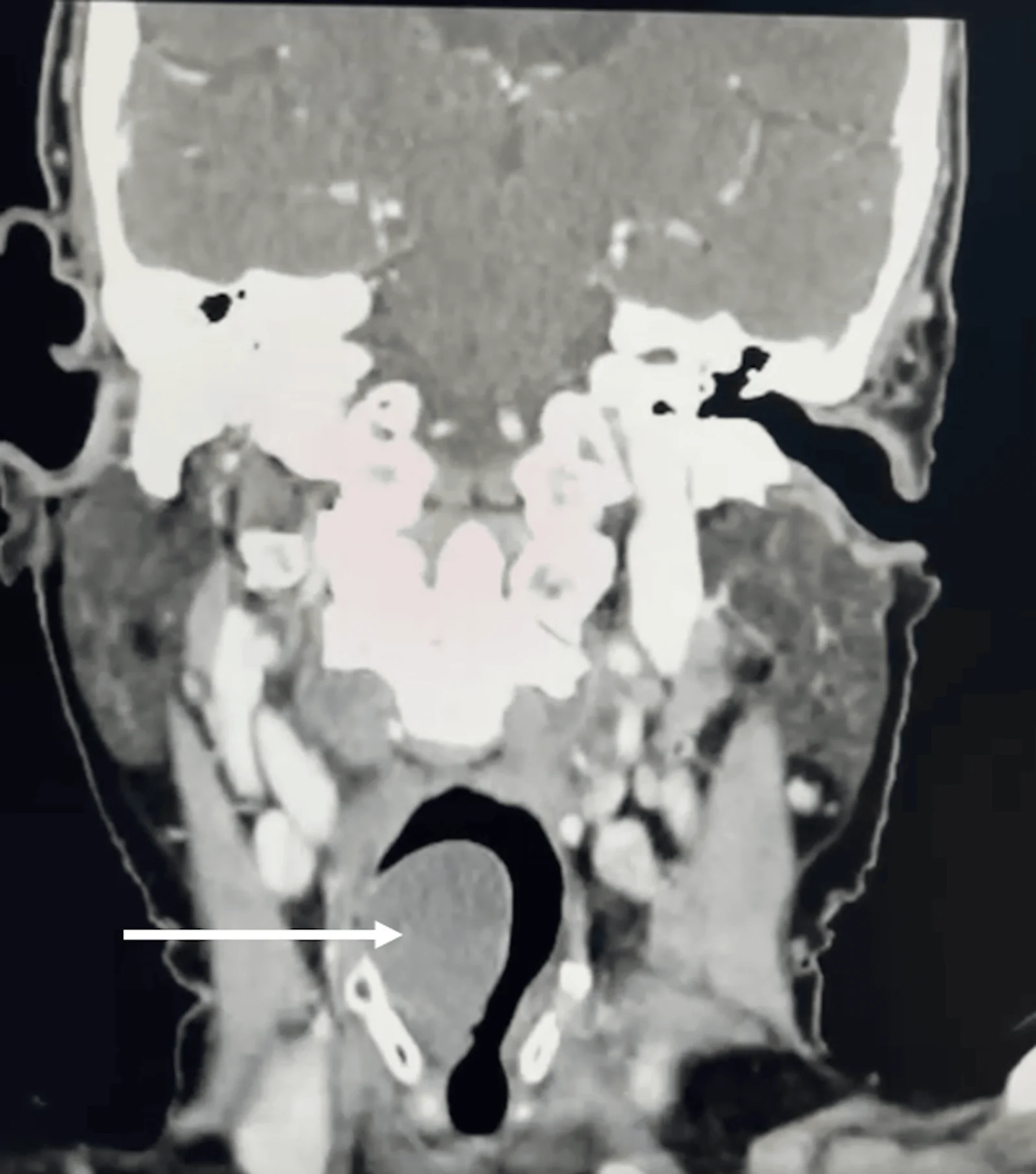
Coronal CT of the neck demonstrating a right-sided, fluid-filled internal laryngocele

She proceeded to undergo microlaryngoscopy and deroofing of the infected right laryngocele. Purulent discharge was expressed upon puncture of the laryngocele wall, confirming the diagnosis of laryngopyocele. Microscopy and culture later only grew normal upper respiratory tract flora. Histopathology confirmed a mucocele with no dysplasia or malignancy.

She was kept intubated postoperatively as a precaution in case of airway swelling. A video laryngoscopy performed the next day showed no reaccumulation of the sac and nil further pus, and she was subsequently extubated uneventfully. High-dose intravenous steroid therapy was continued for 48 hours, and intravenous antibiotic treatment continued for 72 hours, and she was discharged home on postoperative day three on oral antibiotics for a total of seven days. On subsequent follow-up visit at one month postoperatively, she was well recovered with no further airway symptoms. FNE showed that the laryngopyocele site was well healed and mucosalised, with no sign of recurrence.

Case 2

An 89-year-old female patient from a nursing home presented to the emergency department with three weeks of cough and one week of noisy breathing, with an inability to lie flat. She had several months of progressive dyspnoea, dysphagia, hoarse voice, and wet cough with clear sputum. Her general practitioner had previously treated her with an extended course of antibiotics in the community, with little improvement in her symptoms. Her notable past medical history included atrial fibrillation on dabigatran and macrocytic anaemia. 

On examination, she had signs of significant airway compromise with severe dysphonia and intermittent expiratory stridor. She remained stable on room air and afebrile, with brief periods of hypoxia down to an SpO2 of 90%. Her respiratory rate was 22 breaths per minute, blood pressure was 160/70mmHg, and heart rate was 62 beats per minute. FNE revealed a large pedunculated left false vocal cord lesion with a small posterior glottic gap (Figure 3). She received intravenous dexamethasone and cefazolin, as well as idarucizumab for reversal of dabigatran. No imaging studies (e.g., CT or MRI) were done preoperatively.

**Figure 3:**
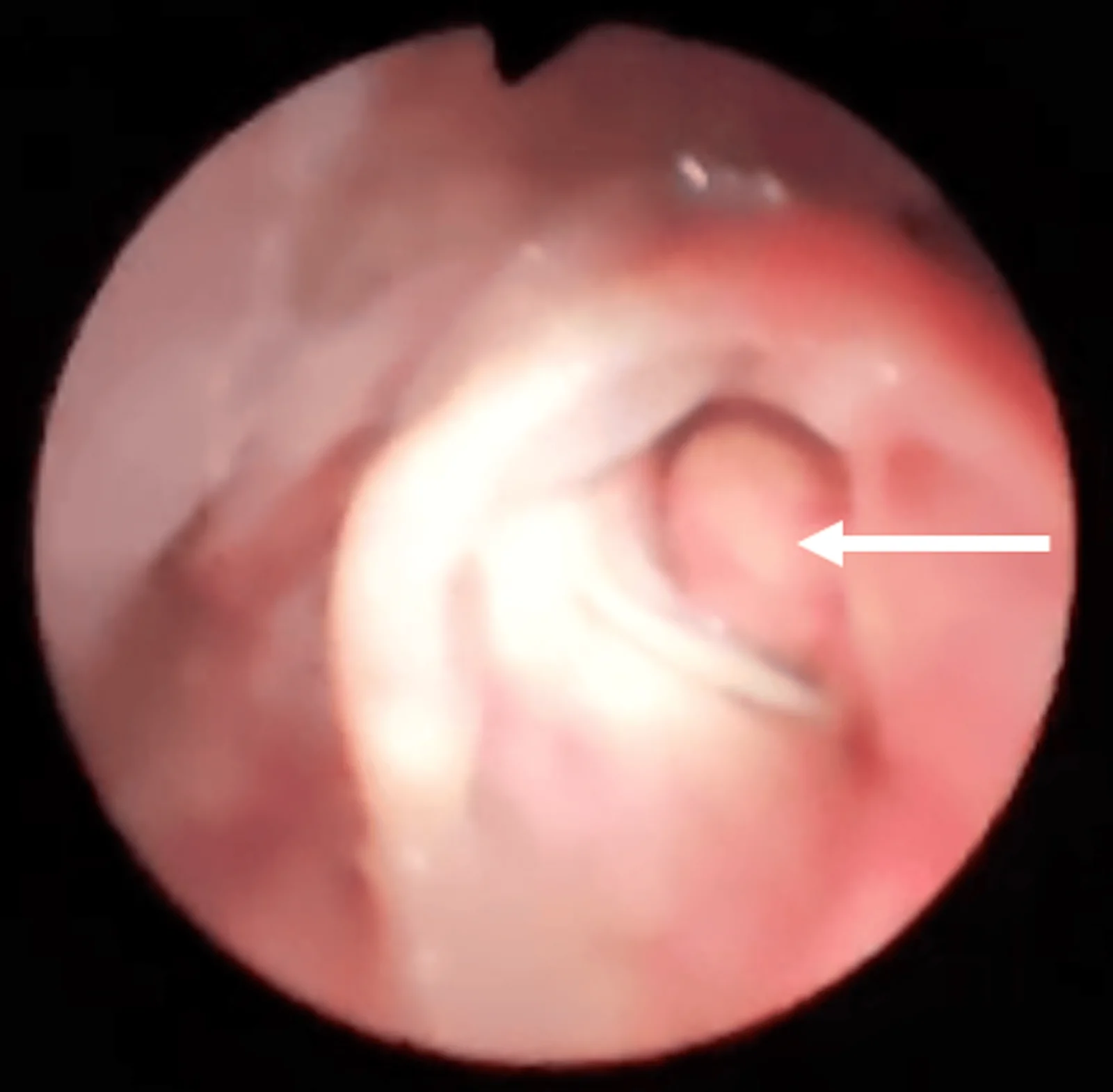
Preoperative FNE image demonstrating a large pedunculated left false vocal cord lesion FNE: Flexible nasendoscopy

She subsequently underwent emergency microlaryngoscopy, tracheostomy and deroofing of infected left laryngocele (Figure 4). Tubeless apnoeic anaesthetic was initially attempted with Optiflow; however, it was abandoned due to poor ventilation and oxygenation, and the patient was intubated with a microlaryngeal tube. The surgical access with the endotracheal tube was limited, and a surgical tracheostomy was placed to allow access and create a safe airway. The laryngocele was deroofed with cold-steel instruments, and copious purulent discharge was expressed, confirming the laryngopyocele (Figure 5). Microscopy and culture results later returned as *Escherichia coli*, and histopathology confirmed a left saccular cyst with no dysplasia or malignancy. 

**Figure 4:**
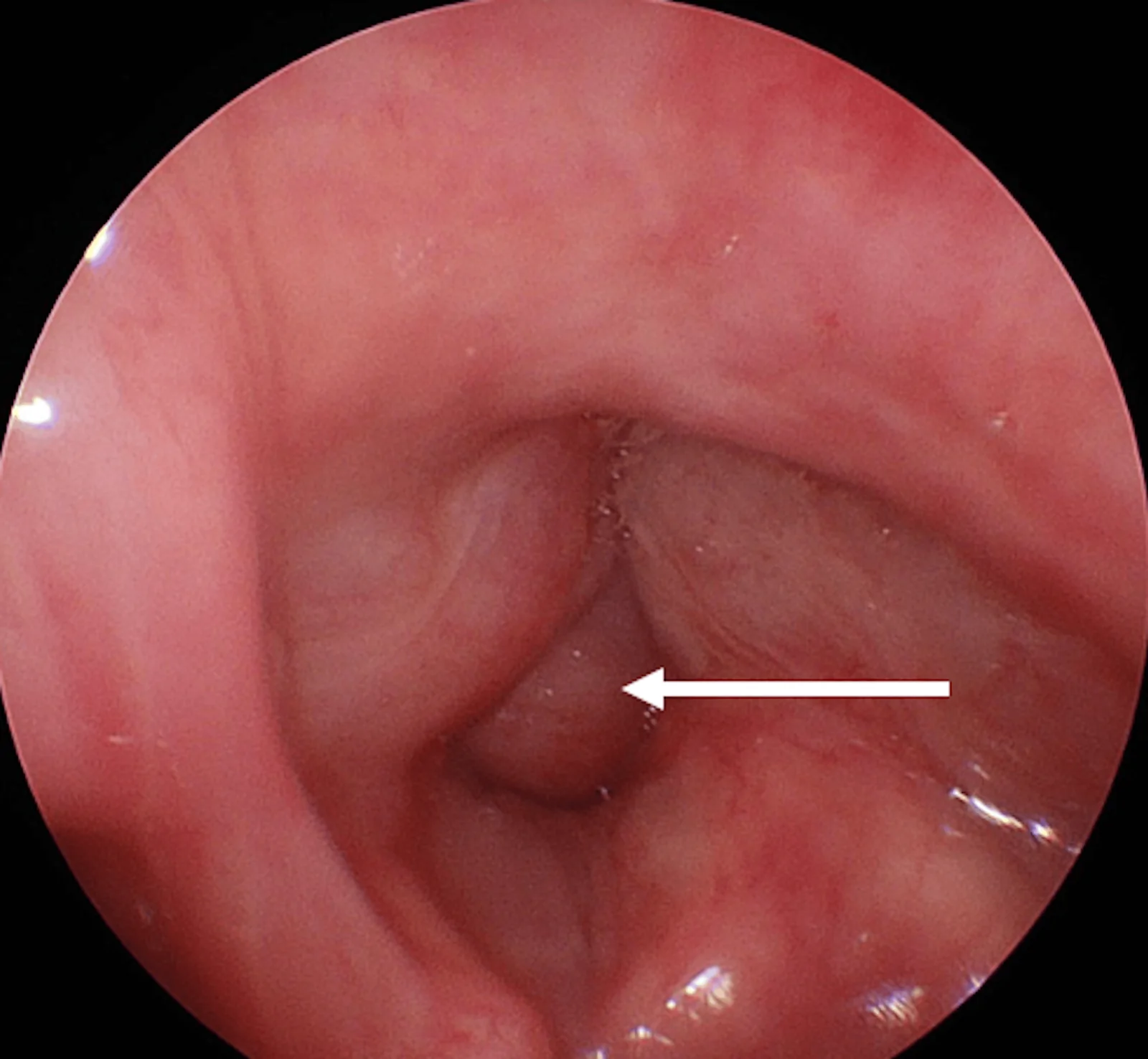
Clinical intraoperative photograph demonstrating a left laryngopyocele

**Figure 5:**
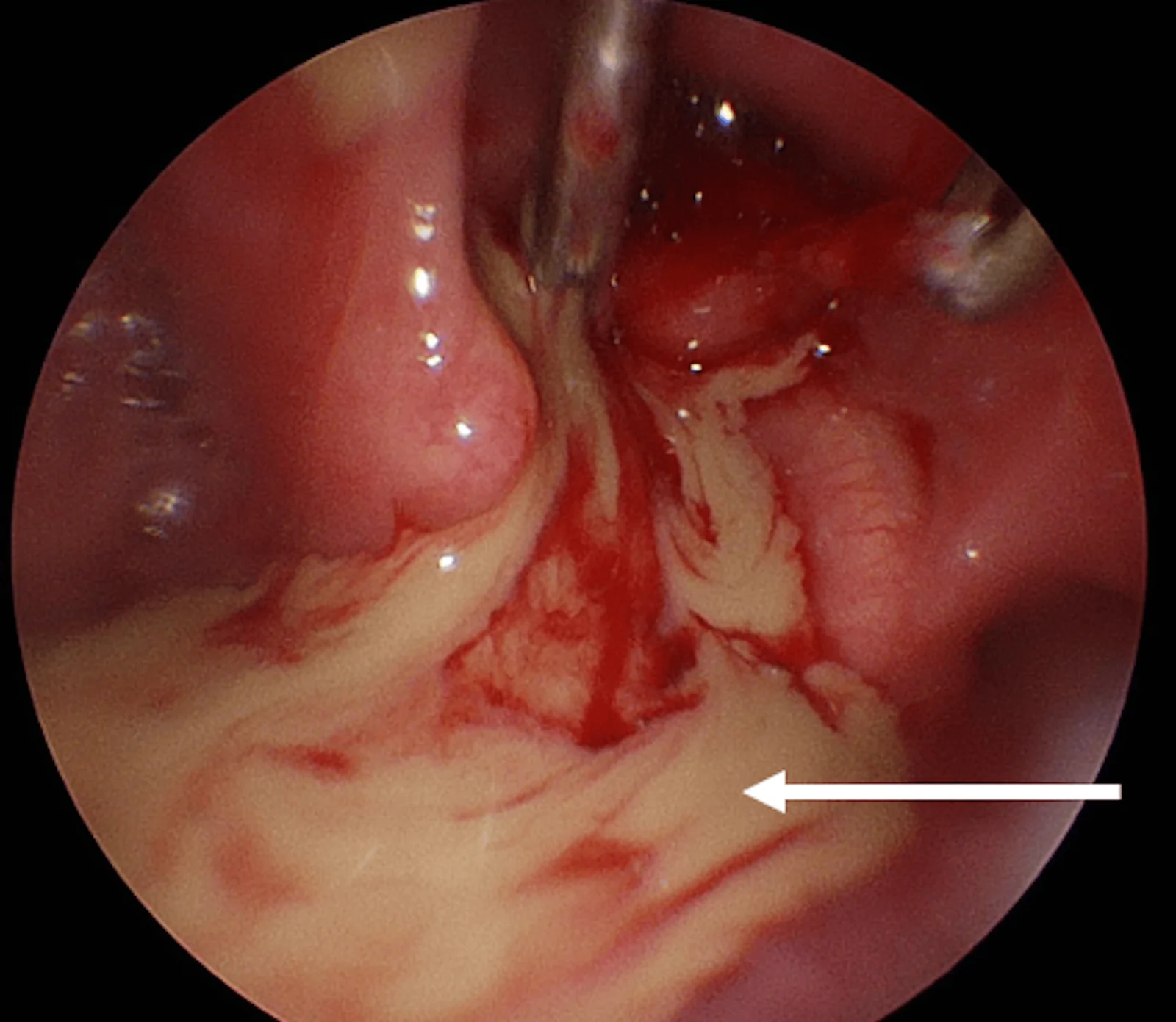
Clinical intraoperative photograph demonstrating copious purulent discharge after deroofing of the laryngopyocele

Postoperatively, the patient received high-dose intravenous dexamethasone for 48 hours. A postoperative CT neck with contrast was performed on postoperative day two, which showed no residual cyst and small volume subcutaneous emphysema on the right neck and gas locules within the glottis, which were thought to be from the recent tracheostomy placement. She was on the wards on postoperative day three and was successfully decannulated on postoperative day six. She was discharged home the next day. Follow-up at one month postoperatively confirmed that she had recovered well and was asymptomatic with no further airway symptoms. FNE showed a normal larynx with no recurrence of laryngocele.

Comparison

Table 1 describes a comparison between the two cases of their presenting symptoms, preoperative workup, intraoperative findings and management, and follow-up outcomes. Both patients presented with an acute-on-chronic progression of their airway symptoms, and preoperative FNE, intraoperative purulent drainage, and postoperative histopathology results supported a diagnosis of laryngopyocele. Case 1 had a CT performed preoperatively, while case 2 did not have a preoperative CT due to her acuity. Both cases were successfully managed with endoscopic drainage, with no signs of recurrence at the one-month follow-up.

**Table 1:** Comparison of case 1 and case 2 FNE: Flexible nasendoscopy

	Case 1	Case 2
Age (years)	90	89
Sex	Female	Female
Presentating symptoms	Six months of progressive dysphonia, worsening over the last month to dysphagia/odynophagia, then acute four-day decompensation with stridor at rest and nocturnal choking	Several months of dyspnoea/dysphagia/hoarse voice/wet cough, then acute three weeks of worsening cough and one week of noisy breathing
SpO2	93% on 2L low-flow nasal prongs	Transiently 90% on room air
Respiratory rate (breaths/minute)	23	22
Blood pressure (mmHg)	143/90	160/70
Heart rate (beats/minute)	82	62
Temperature (degrees Celsius)	36	36.8
Preoperative FNE	Large smooth-walled swelling in the right supraglottis obstructing 75% of the view of the glottis	Large pedunculated left false vocal cord lesion with a small posterior glottic gap
Preoperative imaging	3.6cm right-sided, fluid-filled internal laryngocele, causing 75% narrowing of the airway	N/A
Intraoperative airway management	Awake fibreoptic intubation	Trialled with apnoeic/Optiflow and insertion of a microlaryngeal tube via an operative laryngoscope, but required tracheostomy due to limited access
Intraoperative operative findings	Right laryngopyocele with purulent discharge	Left laryngopyocele with purulent discharge
Microscopy	Normal upper respiratory tract flora	Escherichia coli
Histopathology	Mucocele with no dysplasia or malignancy	Left saccular cyst with no dysplasia or malignancy
Postoperative follow-up time	1 month	One month
Postoperative follow-up FNE	Well healed and mucosalised laryngopyocele site, with no recurrence	Normal larynx with no recurrence of laryngocele

## Discussion

A laryngocele is a rare pathological air-filled dilatation or herniation of the laryngeal saccule, which communicates with the lumen of the larynx [6-11]. The saccule, or the appendix of the laryngeal ventricle of Morgagni, is situated between the ventricular folds, the base of the epiglottis, and the inner surface of the thyroid cartilage [12]. It can vary in size and is lined by respiratory epithelium [13]. The incidence of laryngoceles is unknown but is estimated to affect 1 in 2.5 million people per year [11,14,15]. They usually occur in the sixth decade of life, with male patients reported to be 5-7 times more likely to be affected than female patients [6,9,12-14]. 

Laryngopyocele is a rare and potentially life-threatening complication of a laryngocele that becomes infected and filled with pus [12]. It is estimated that approximately 8% of laryngoceles become infected, and one systematic review by Singh et al. found that there have been only 64 reported cases of laryngopyoceles in the literature from 1925 to 2019 [1-5]. It should be noted that the majority of data on laryngopyoceles comes from small, retrospective, decades-spanning literature reviews (mostly single-institution or pooled case reports), not registries or epidemiological studies. 

Laryngopyoceles occur when chronic inflammation causes stasis of glandular secretions, obstructing the neck of the laryngocele, resulting in laryngomucocele, which can then become infected, become pus-filled, and form a laryngopyocele [3,6,9]. The common pathogens involved are *Staphylococcus aureus*, haemolytic *Streptococcus*, *Escherichia coli*, and *Pseudomonas aeruginosa* [10,12].

The exact aetiology of laryngoceles is unknown, but they are thought to either be congenital, acquired, or both [3,9,12-15]. Congenital laryngoceles are believed to be due to a congenital saccular dilatation of the ventricular appendage that is a remnant of a structure normally found in apes [13,15]. Acquired laryngoceles are thought to be related to risk factors that raise intraglottic pressure, such as chronic coughs, glass blowers, weight lifters, and wind or brass instrument players [10-12]. The straining nature of these activities fixes the diaphragm in forced expiration against a closed larynx, increasing the intralaryngeal pressure and hence increasing the likelihood of developing a laryngocele [15]. However, the rarity of this condition and the fact that they are usually only unilateral suggest that there are also other factors that predispose affected patients [13]. Other factors that have been associated with laryngocele development include laryngeal tumours (which have been reported in up to 18% of laryngocele cases), intravenous drug injection into the neck, laryngeal trauma including vocal cord surgery, and systemic disease including scleroderma [3,6,9,11,12]. Age-related weakening of the thyroepiglottic muscles and increased laxity of the thyrohyoid membrane have also been thought to increase the likelihood of laryngoceles [3].

Laryngoceles are classified based on whether they are confined to the larynx or whether they herniate through the thyrohyoid membrane [4,8]. Internal laryngoceles (approximately 20% of laryngoceles) are confined within the paraglottic space between the false vocal cord and thyroid cartilage, medial to the thyrohyoid membrane, while external laryngoceles (30% of laryngoceles) extend into the neck through the thyrohyoid membrane at the level of the hyoid bone [3,12]. The remaining 50% of laryngoceles are considered mixed, as they have both internal and external components [3]. For laryngopyoceles, Al-Yahya reported in their systematic review that 78% (18/23 included cases) are mixed, 13% (3/23 cases) are internal, and 9% (2/23 cases) are external [1].

The clinical presentation of laryngoceles and laryngopyoceles can vary depending on their size or extension into the laryngeal lumen, with most cases of laryngoceles being asymptomatic [4,10]. If symptomatic, patients may report dysphonia and hoarseness, dysphagia, odynophagia, neck pain or a foreign body sensation in the throat [3,9,12]. External laryngoceles may present as an anterolateral compressible neck lump, which increases in size upon Valsalva manoeuvres [12,15]. In cases of laryngopyoceles, patients may also present with stridor and sepsis [3]. Rarely, they can be an unusual cause of acute airway obstruction. There have been at least 11 previously documented reports of laryngopyocele cases presenting with acute airway obstruction, with Raine et al. describing the eleventh case in 2014 [2].

Diagnosis involves a combination of imaging studies and endoscopic evaluation. FNE usually shows a lump on the ventricle and fullness of the false vocal cord, aryepiglottic fold or pyriform sinus, which may obscure the view of the glottis [3,12]. CT scan is considered the most accurate diagnostic modality, as it will aid in delineating between laryngopyocele and deep neck space infections, as well as allow operative planning by assessing its relationship with the inner laryngeal structure [7,10]. Laryngoceles usually appear as an air-filled supraglottic mass originating in the larynx, with possible air-fluid levels in laryngopyoceles [12]. MRI can be used to better visualise soft tissue structures as well as to assess for any concurrent malignancies, although this can be limited by the patient’s ability to lie flat for prolonged periods without a secured airway. Ultrasound can also be used to characterise general neck lumps for potential aspiration, but CT is usually the preferred modality if the patient is unwell and further surgical planning is required [3,12].

The initial treatment priority is to evaluate and secure the airway. This can include oxygen, nebulised adrenaline and systemic steroids, as well as endotracheal intubation or tracheostomy if required [2,13,14]. If the patient is septic, they should be managed according to the sepsis six principles and commenced on broad-spectrum antibiotics [3]. This is particularly important in cases of laryngopyoceles, which have a wide range of potential microorganisms involved. For the laryngopyocele, the mainstay of treatment is surgical drainage [9]. Some authors suggest that ultrasound-guided aspiration is an acceptable initial measure, followed by definitive excision at a later date [12,13]. Others suggest that complete excision can be achieved at the same time as the initial surgery [14]. While there is one documented case of a spontaneously regressing laryngopyoceles after antibiotic treatment [16], there is still the general consensus that drainage followed by formal excision at some stage is required in order to prevent reaccumulation and the associated potentially life-threatening complications. The aspirated/drained purulent contents should be sent for microscopy and culture to guide antibiotic treatment, and the laryngocele should also be sent for histology to rule out malignancy. The patient should also be followed up after their recovery from the acute infection to exclude a synchronous malignancy.

The surgical approach for laryngoceles depends on their type. For external and mixed laryngoceles, an open approach via transverse neck incision at the level of the thyrohyoid membrane is generally performed [3,10]. Internal laryngoceles can be managed with an endoscopic approach to decompression via microlaryngoscopy [12]. Endoscopic advancements have allowed increased instrumentation options, including cold instrumentation, microdebrider, and CO2 laser [1]. Singh et al. also reported that transoral robotic surgery is an emerging treatment modality in recent times, although microlaryngoscopy still remains the gold standard for internal laryngoceles [5]. 

Complications of untreated laryngopyoceles can be fatal, and there have been at least three documented mortalities from laryngopyocele cases between 1954 and 2015 [17]. The most common complication is airway obstruction from mass effect [3,12]. Laryngopyoceles can also spontaneously rupture, causing purulent discharge to be spilled into the airways or parapharyngeal spaces, which can lead to aspiration, jugular venous thrombosis, or mediastinal abscess [5,12].

## Conclusions

We highlight the successful endoscopic management of two elderly patients with laryngopyoceles. The rarity of laryngopyoceles highlights the importance of maintaining a high index of suspicion in patients presenting with acute-on-chronic signs of airway compromise or potential deep neck space infection. Suspected cases should usually undergo prompt flexible nasal laryngoscopy evaluation with an early ENT referral. Preoperative CT is valuable when the patient's condition allows, though acute airway compromise may necessitate proceeding directly to surgical intervention, such as in the second case. Consideration should also be given to the most suitable approach to managing the airway and surgical approach to drainage and excision. Early recognition and definitive management of laryngopyoceles are important to prevent potentially life-threatening complications, and the two patients were found to have no postoperative recurrence or complications at the one-month follow-up following endoscopic laryngeal surgery.
